# Subtyping of STEC by MLVA in Argentina

**DOI:** 10.3389/fcimb.2012.00111

**Published:** 2012-08-22

**Authors:** Ana V. Bustamante, Andrea M. Sanso, Alberto E. Parma, Paula M. A. Lucchesi

**Affiliations:** ^1^Laboratorio de Inmunoquímica y Biotecnología, Facultad de Ciencias Veterinarias, Universidad Nacional del Centro de la Provincia de Buenos AiresTandil, Argentina; ^2^Consejo Nacional de Investigaciones Científicas y Técnicas, Buenos AiresArgentina

**Keywords:** STEC, MLVA, genotyping, O157:H7, non-O157

## Abstract

Shiga toxin-producing *Escherichia coli* (STEC) causes serious human illness such as hemolytic uremic syndrome (HUS). Argentina has the world’s highest rate of this syndrome, which is the leading cause of acute renal failure among children. *E. coli* O157:H7 is the most common cause of HUS, but a substantial and growing proportion of this illness is caused by infection due to non-O157 strains. Multiple-locus variable-number tandem repeat analysis (MLVA) has become an established technique to subtype STEC. This review will address the use of routine STEC subtyping by MLVA in order to type this group of isolates and to get insight into the genetic diversity of native STEC. With regard to these objectives we modified and adapted two MLVA protocols, one exclusive for O157 and the other, a generic *E. coli* assay. A total of 202 STEC isolates, from different sources and corresponding to 20 serotypes, have been MLVA genotyped in our laboratory. In our experience, MLVA constitutes a very sensitive tool and enables us to perform an efficient STEC subtyping. The diversity found in many serotypes may be useful for future epidemiological studies of STEC clonality, applied to O157 as well as to non-O157 isolates.

higa toxin-producing *Escherichia coli* (STEC), also called verotoxin-producing *E. coli* (VTEC), is the most important recently emerged group of foodborne pathogens. STEC can produce serious human illness linked to the consumption of contaminated foods, mainly of bovine origin. Argentina has the highest rate of hemolytic uremic syndrome (HUS) globally and HUS is the leading cause of acute renal failure among children (Cobeñas et al., 2007). *E. coli* serotype O157:H7 is the most common cause of HUS, but a substantial and growing proportion of this illness is caused by infection due to non-O157 strains (Johnson et al., 2006).

acterial typing methods generate strain specific molecular fingerprints to assess the epidemiological relationship among isolates. Lately, our laboratory is focused on implementing multiple-locus variable-number tandem repeat analysis (MLVA) as genotyping method, which will be reviewed here. With regard to this objective we modified and adapted two previously described MLVA protocols, one exclusive for O157:H7 (Lindstedt et al., 2003; Keys et al., 2005; Noller et al., 2006), based on polymorphism in nine variable number of tandem repeats (VNTR) loci – named MLVA_O157_ in the present manuscript (Bustamante et al., 2009a), and the other, a generic *E. coli* assay (Lindstedt et al., 2007) based on seven VNTR loci – named MLVA_G_ (Bustamante et al., 2010).

 total of 202 STEC isolates, tested previously for selected virulence factors, has been MLVA genotyped in our laboratory between 2006 and 2010. They have been isolated from bovines, foods, and patients in previous studies (Parma et al., 2000; Padola et al., 2004; Sanz et al., 2007; Fernández et al., 2010a; Rivero et al., 2010). Twenty-eight isolates belonged to O157:H7 serotype and the remaining ones were non-O157:H7 belonging to 19 serotypes. In order to name the VNTR alleles we used a nomenclature where the actual number of repeats at each locus is reported. Alleles which presented partial repeats were rounded to the nearest complete repeat number. If no amplification product was detected, the allele was designated with an arbitrary number (30). All genotyping data were stored as allelic number strings which is an easy way of comparing isolates.

n the case of MLVA_O157_, we carried out twodifferent studies. In the first one, we analyzed a set of 15 STEC O157:H7 mostly isolated from cattle. The isolates could be grouped according to MLVA profiles in two main clusters, one that grouped all the bovine isolates from the same farm and the other one, the rest of the isolates. Within the cluster of STEC isolated from the same farm (*n* = 10) it was possible to identify four profiles which shared alleles for two loci (TR3 and O157-37). In concordance with the origin of the samples, the differences between unrelated isolates were greater than those presented by isolates from the same farm (Bustamante et al., 2009a).

 second study included 13 STEC O157:H7 of human origin. They have been isolated from children with diarrhea and/or HUS living in Tandil and its surroundings (province of Buenos Aires). We detected as many profiles as examined isolates, which highlights a great O157:H7 genetic diversity in a same geographic region (Rivero et al., 2008). There were no epidemiological associations between the isolates from the first and the second studies and, as expected, the profiles obtained in the second were different from those of the first one. Taking into account both studies, the results revealed 22 different profiles, from which 20 were unique. A similar proportion was detected by other authors (Lindstedt et al., 2003) studying a larger number of samples. We observed variation at all nine loci and the most variable locus was TR2, coincidently with the results of Hyytiä-Trees et al. (2006) and Noller et al. (2006).

n relation with the MLVA_G_ we applied it in order to analyze both non-O157:H7 and O157:H7 isolates (Bustamante et al., 2009b, 2010; Fernández et al., 2010b; Franci et al., 2011). In a total of 174 samples we detected 66 (37.9%) different MLVA profiles, being 41 of them unique. To our knowledge, we subtyped by MLVA for the first time 14 out of the 20 serotypes studied: O8:H19, O20:H19, O91:H21, O112:H2, O113:NM, O117:H7, O130:H11; O145:NM, O171:H2, O174:H21, O171:NM, ONT:H7, ONT:H19, and ONT:H21. Also, we observed several alleles which have not been previously described. The locus CVN014 was the most variable among serotypes and among isolates from a same serotype (Table 1), coincidently with the results of Lindstedt et al. (2007) and Gorgé et al.(2008). Among non-O157:H7 serotypes, the loci which presented the lowest variability were CVN002, CVN007, CVN015, and CVN003. Furthermore, this last locus presented null alleles (no PCR amplification) in all isolates except for those belonging to O157:H7 and O145:NM serotypes (Bustamante et al., 2010). Similarly, Løbersli et al. (2012) found this locus was absent in several serotypes and they only confirmed the presence of this locus among *E. coli* O145, O157, and O55:H7 isolates.

**Table 1:** Alleles detected by MLVAG: distribution by serotype and locus.

Serotypes	Loci						
	*CVN001*	*CVN002*	*CVN003*	*CVN004*	*CVN007*	*CVN014*	*CVN015*
08:H19	7 9	1	NA	12	6	9, 10, 11, 12	5
O20:H19	7 9	1	NA	10, 12	6	7 11, 16	5
022:H8	7	1	NA	12	6	7	5
091:H21	7	1	NA	12	6	5, 6, 7	5
0112:H2	7	1	NA	12	6	8	5
0113:H21	7	1	NA	9, 12	NA, 6	6, 7 9, 10	NA, 5
0113:NM	7	1	NA	12	NA, 6	8	NA, 5
0117: H7	7	1	NA	12	6	6, 8, 10, 11	5
0130: H11	5	2	NA	9	8	NA	6
0145:NM	8	1	2	12	6	3	5
0157:H7	7	5, 7 8, 9	3, 4, 5	10, 13	8, 9, 10	5, 6, 8, 9, 10, 11	8, 10
0171:H2	1, 8	1	NA	12	6	2, 6, 8, 9, 10, 11	5
0171:NT	8	1	NA	12	6	7	5
0171:NM	1	1	NA	12	6	7	5
0174:H21	6, 7, 8, 9	1	NA	12	6	5, 7 8, 9, 11, 12, 14, 15, 16, 18	5
0178:H19	7 9	1	NA	11, 12	6	7 14, 15	5
0NT:H7	7 9	1	NA	12	6	5, 9, 10	5
0NT:H19	9	1	NA	12	6	5	5
0NT:H21	7	NA	NA	12	6	8, 11	5
0NT:NT	7 9	1	NA	12	6	5, 7 10	5

*NA, null allele.*

The results obtained performing MLVA_O157_ and MLVA_G_ showed a high genetic diversity in the STEC isolates analyzed, and five or more MLVA profiles were found in the serotypes O20:H19, O117:H7, O157:H7, O171:H2, O174:H21, and O178:H19 (Figure 1). On the contrary, preliminary data in regard to O130:H11 serotype, showed a unique profile in all the studied isolates which could be indicating that it is an emergent serotype or, on the contrary, that the chosen VNTR loci are not variable enough (Fernández et al., 2010b).

**FIGURE 1:**
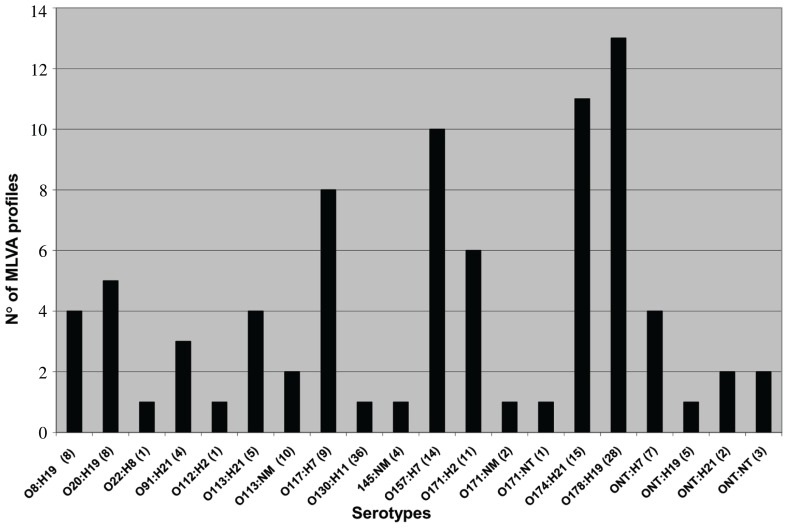
**Number of MLVA profiles obtained from each serotype typed by MLVA_G_**. Number of isolates is indicated between parentheses.

Another laboratory from Argentina has begun to evaluate the use of the MLVA for the epidemiological surveillance of *E. coli* O157:H7, as a complementary technique to pulsed-field-gel-electrophoresis (PFGE) in order to solve difficult cases (Chinen et al., 2010). The chosen protocol implies the study of eight VNTRs described by Hyytiä-Trees et al.(2010), some of which are also analyzed in the MLVA_O157_. Using that MLVA approach they were able to distinguish between sporadic cases and outbreaks, with higher discrimination than PFGE. Other authors who also applied MLVA for STEC typing obtained a higher number of MLVA than PFGE profiles and observed that MLVA was particularly useful to discriminate epidemiologically unrelated isolates (Keys et al., 2005; Hyytiä-Trees et al.2010; Izumiya et al., 2010; Konno et al., 2011).

LVA_G_ worked well with the majority of STEC serotypes. However, in the case of some serotypes it was not possible to discriminate enough and, in consequence, this method could be improved by incorporating more loci. Recently, Løbersli et al. (2012) improved that method by adding three new repeat-loci to a total of 10. They applied it and observed a considerable increase in resolution, of 71%, using the three new loci. Now, we are in process of adapting this proposed method in our laboratory and using it to subtype STEC. Regarding O157:H7 serotype, both MLVA protocols allowed to find high genetic diversity. In addition, they showed variability in all the VNTR loci analyzed in the MLVA_O157_. This protocol was the one that better reflected the epidemiological relationship among the isolates.

## CONCLUDING REMARKS

n our experience, MLVA works well at our laboratory and enables us to perform an efficient O157:H7 and non-O157 STEC subtyping. The obtained results showed a high genetic diversity in the analyzed STEC isolates. The approach also allowed us to stablish possible associations between MLVA genotypes and parameters such as source and virulence characteristics. The diversity found in many serotypes may be useful for future epidemiological studies of STEC strains, of both O157 as well as non-O157 serogroups.

## Conflict of Interest Statement

he authors declare that the research was conducted in the absence of any commercial or financial relationships that could be construed as a potential con- flict of interest.
